# Supplementary figures and images for: Comparison of Root Transcriptomes against Clubroot Disease Pathogens in a Resistant Chinese Cabbage Cultivar (Brassica rapa cv. ‘Akimeki’)
Source: Plants (Basel). 2024 Aug 5;13(15):2167. doi: 10.3390/plants13152167 (PMC11314269; doi:10.3390/plants13152167)

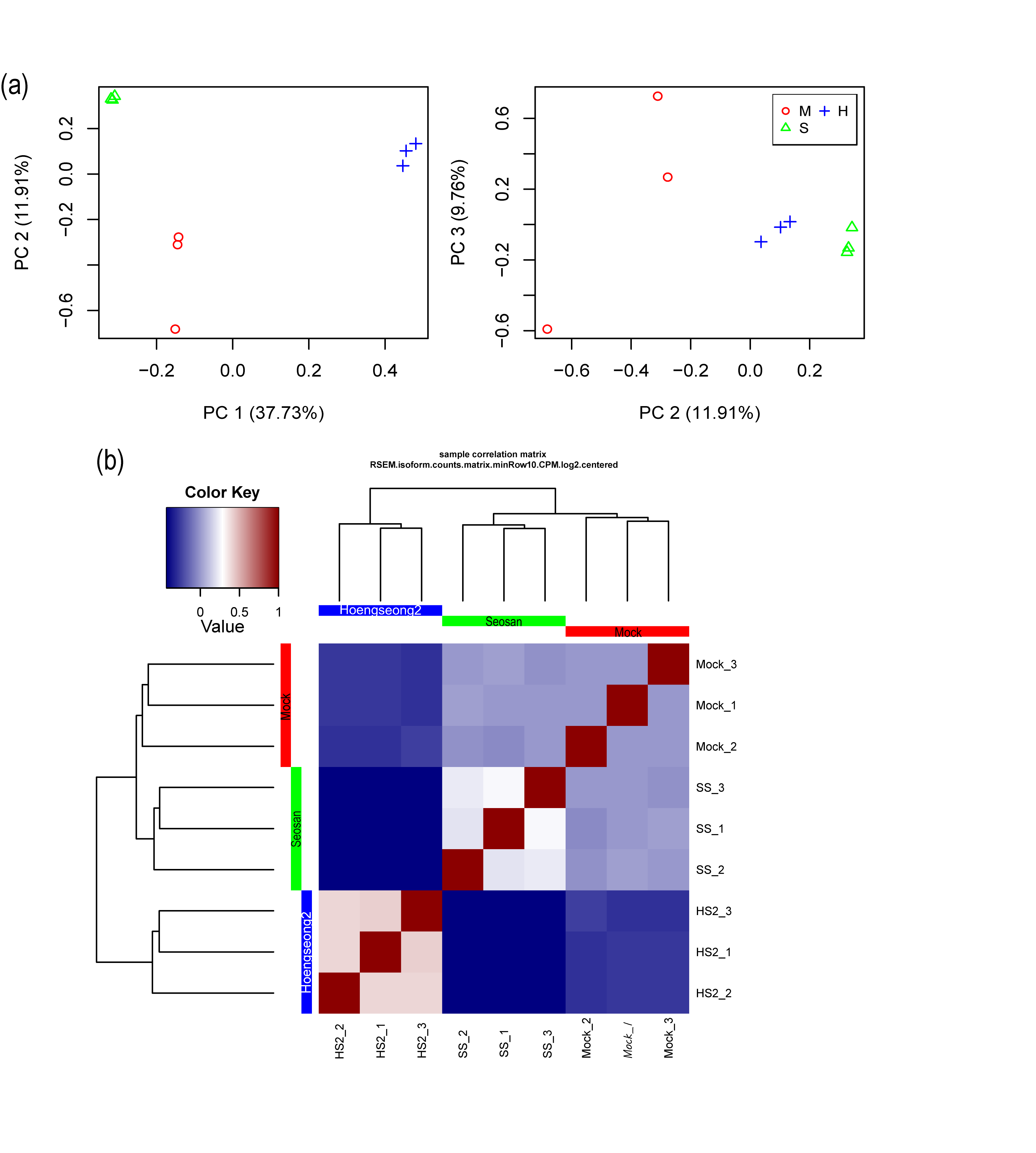

Supplement: Supplementary file 1 [file plants-13-02167-s001.zip › Supplementary_Figure S1.PCA_and_correlation_analysis.tif]

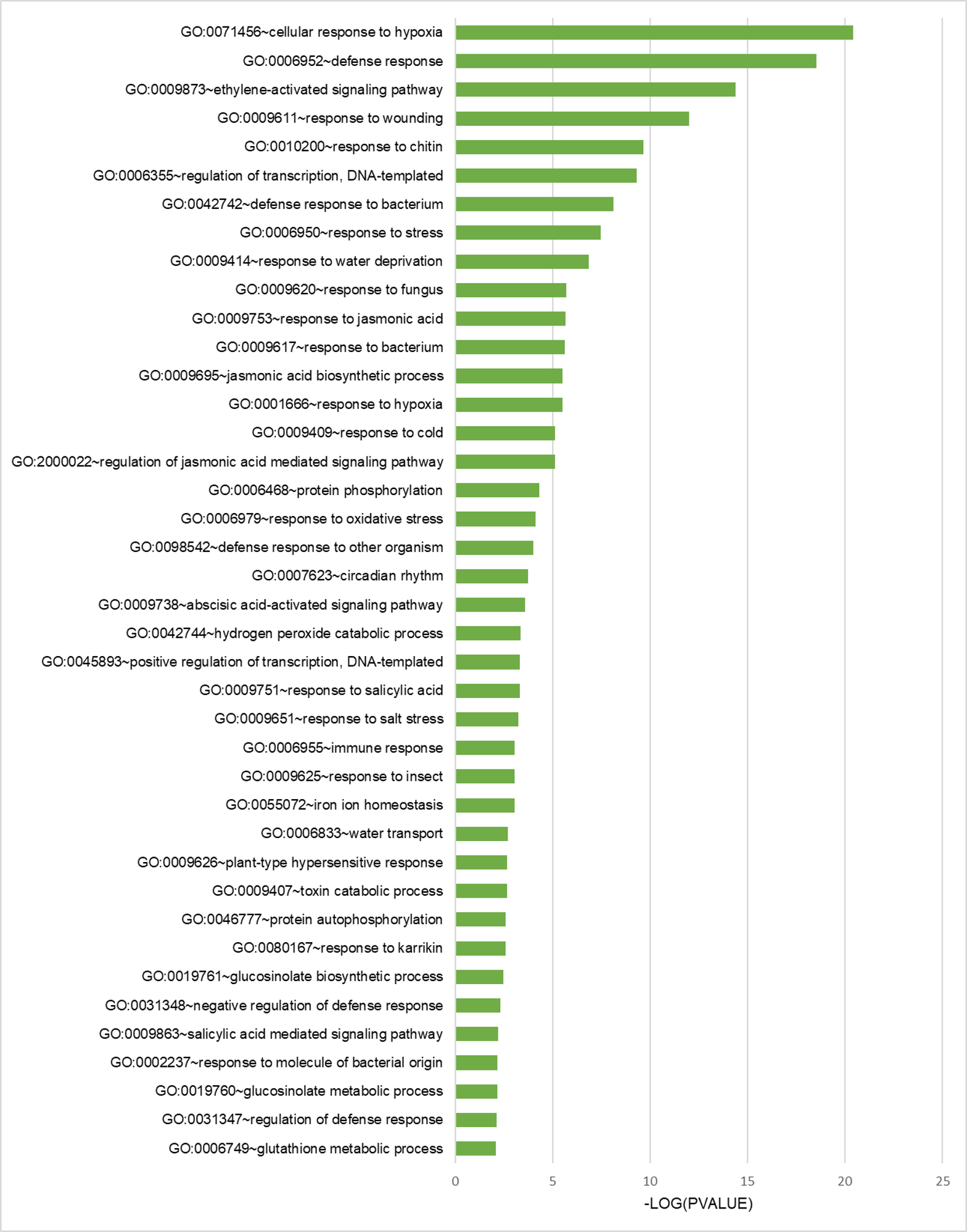

Supplement: Supplementary file 1 [file plants-13-02167-s001.zip › Supplementary_Figure S2.Gene Ontology (GO) analysis of the 2037 DEGs between SS inoculation and HS2 inoculation.tif]
